# A potential prognostic lncRNA signature for predicting survival in patients with bladder urothelial carcinoma

**DOI:** 10.18632/oncotarget.14441

**Published:** 2017-01-02

**Authors:** Zhenyu Bao, Weitao Zhang, Dong Dong

**Affiliations:** ^1^ Shanghai Key Laboratory of Regulatory Biology, Institute of Biomedical Sciences, School of Life Sciences, East China Normal University, Shanghai, China; ^2^ Urology Surgery Department, Affiliated Hospital of Taishan Medical University, Shandong, China

**Keywords:** long non-coding RNA, prognosis, bladder urothelial carcinoma

## Abstract

Increasing evidence has highlighted the critical roles of long non-coding RNA (lncRNA) in cancer development and progression. However, the prognostic power of expression-based lncRNA signature for predicting overall survival in patients with Bladder Urothelial Carcinoma (BLCA) has not been investigated. Here, we performed a comprehensive analysis for lncRNA expression profiles and corresponding clinical information of 234 BLCA patients from The Cancer Genome Atlas (TCGA). We established a set of four-lncRNAs that were significantly associated with BLCA patients’ survival. Using the prognostic four-lncRNA signature, we successfully classified the BLCA patients into high-risk and low-risk groups, and the prognostic power of the four-lncRNA signature was further validated in the testing dataset and entire dataset. Multivariate Cox regression and stratified analyses demonstrated that the prognostic power of the four-lncRNA signature was independent of other clinical variables. Functional enrichment analyses suggested the four prognostic lncRNAs may be involved in known BLCA-related biological processes and pathways. Our results demonstrated that the four-lncRNA signature could be novel independent biomarkers for predicting survival in patients with BLCA.

## INTRODUCTION

Bladder cancer is the ninth most common malignancy worldwide [1]. It has been estimated about 80000 newly diagnosed bladder cancer cases in the United States in 2015 [2]. Bladder Urothelial Carcinoma (BLCA) is the most common histological subtype of bladder cancer. Overall, about 70% of bladder tumors are non-muscle-invasive bladder cancer, and the others are muscle-invasive bladder cancer [3]. Despite recent advances in the surgical technique, the overall survival of BLCA patients has not been dramatically improved, and the five-year survival rate remains at only 50-60% [4–6]. Therefore, it is necessary to identify novel independent biomarkers for diagnostic and prognosis and to develop new targeted therapies for BLCA patients.

Long non-coding RNAs (lncRNAs) are an important category of non-coding RNAs (ncRNAs) with little or no protein-coding capacity, which range from 200 nucleotides to multiple kilobases in length [7, 8]. Accumulated evidence suggests that lncRNAs play crucial roles in regulating gene expression at transcriptional, posttranscriptional and epigenetic levels [7, 9], and participate in various biological processes and pathways, such as transcriptional regulation, cell growth and tumorigenesis [10, 11]. Like mRNA and miRNA, some well-studied lncRNAs have been found to play critical oncogenic or tumor suppressive roles in various types of cancers [12–14]. For instance, *HOTAIR*, *MALAT1* and *CRNDE* have been showed as oncogenic roles [15–25], while *GAS5*, *MEG3* and *lincRNA-p21* as tumor suppressive roles [26–32]. Currently, several expression-based lncRNA signatures have been identified in glioblastoma [33], oesophageal squamous cell carcinoma [34], breast cancer [35], colorectal cancer [36], non-small cell lung cancer [37], multiple myeloma [38] and ovarian cancer [39], highlighting their potential roles as novel independent biomarkers for cancer prognosis. For bladder cancer, recent studies have also revealed that lncRNAs (*HOTAIR*, *SPRY4-IT1*, *SUMO1P3* and *PANDAR*) are aberrantly expressed in BLCA patients [40–43]. However, the prognostic power of expression-based lncRNA signature for predicting BLCA patients’ survival remains unclear.

In this work, we performed a comprehensive analysis for lncRNA expression profiles and corresponding clinical information of BLCA patients in the TCGA training dataset. We identified four lncRNAs significantly associated with patients’ survival and constructed a four-lncRNA signature that can effectively predict patients’ survival. The testing dataset and entire dataset further validated the prognostic power of the four-lncRNA signature. Our results demonstrated the four-lncRNA signature can function as novel independent biomarkers for BLCA prognosis and provide novel insights into understanding of the underlying molecular mechanism of BLCA.

## RESULTS

### Identification of prognostic lncRNAs associated with patients’ survival from the training dataset

The 234 patients with BLCA were randomly divided into a training dataset (*n* = 117) and a testing dataset (*n* = 117). The training dataset was analyzed to identify prognostic lncRNAs. At first, we performed a univariate Cox regression analysis to evaluate the association between the expression profiles of each lncRNA and patients’ survival in the training dataset. The result showed that four lncRNAs were identified as prognostic lncRNAs (*p-value* < 0.001). The detailed information of these four lncRNAs was showed in Table 1. Among these prognostic lncRNAs, the lncRNAs (*AC005682.5* and *CTD-2231H16.1*) with higher expression profiles were associated with shorter survival (coefficient > 0), while the remaining two lncRNAs (*CTB-92J24.2* and *RP11-727F15.13*) with higher expression profiles were associated with longer survival (coefficient < 0).

**Table 1:** The detailed information of four prognostic lncRNAs significantly associated with overall survival in patients with BLCA

Ensembl ID	Gene symbol	Chromosomal position	*P* value^a^	Hazardratio^a^	Coefficient^b^
ENSG00000228649	*AC005682.5*	chr7: 22,854,178-22,861,579 (+)	3.39E-04	1.613	0.371
ENSG00000249430	*CTD-2231H16.1*	chr5: 92,151-139,863 (+)	8.44E-04	0.175	0.175
ENSG00000269397	*CTB-92J24.2*	chr19: 23,927,788-23,929,287 (+)	2.22E-04	0.609	-0.251
ENSG00000269463	*RP11-727F15.13*	chr11: 62,807,682-62,808,063 (-)	6.88E-04	0.736	-0.232

^a,b^Derived from the univariate and multivariate Cox regression analyses in 117 patients of the training dataset.

### Construction and validation of a four-lncRNA signature for predicting patients’ survival in the training dataset

**Figure 1:**
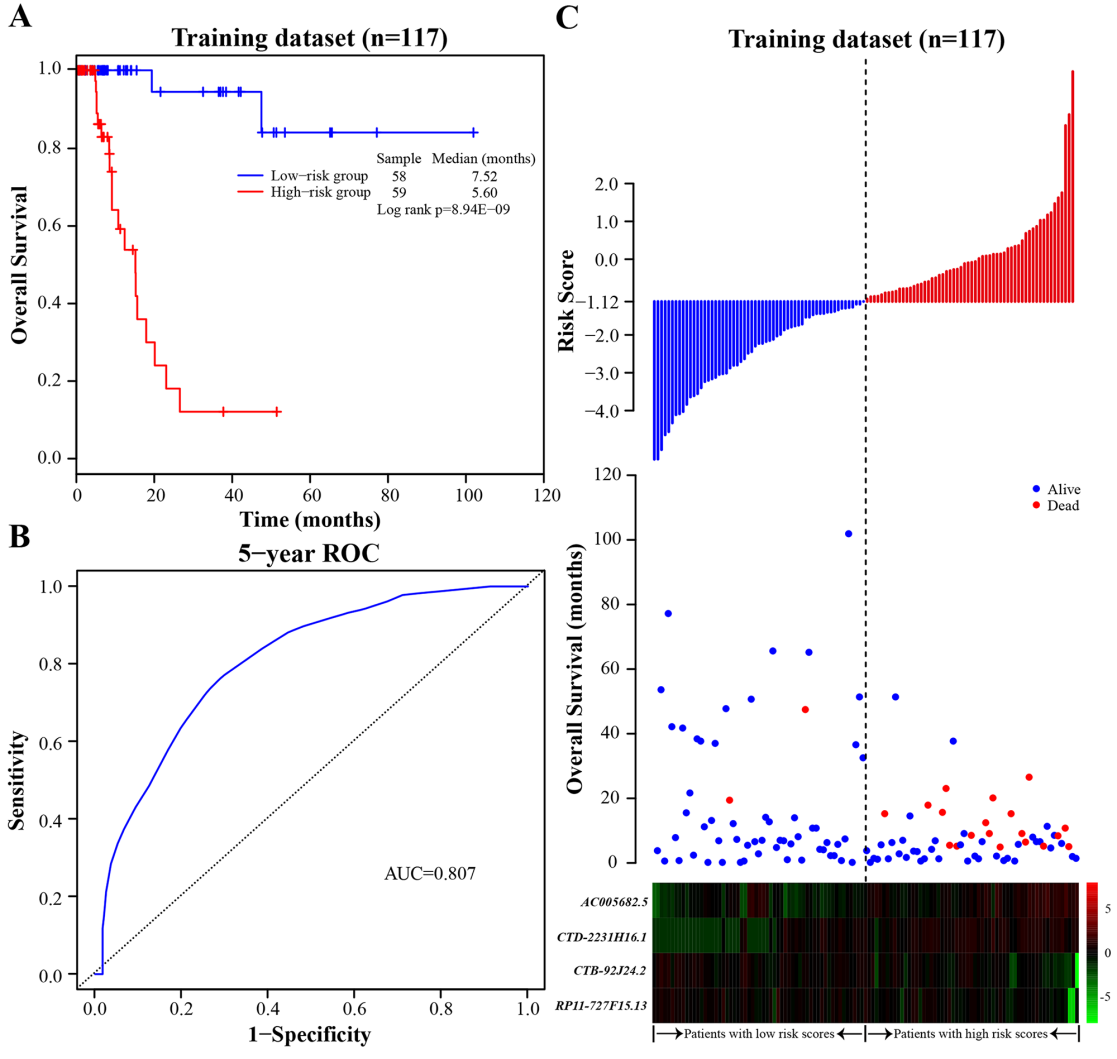
The four-lncRNA signature in prognosis of overall survival of BLCA patients in the training dataset **A**. The Kaplan-Meier curves of overall survival between high-risk and low-risk patients in the training dataset. **B**. The ROC curve for survival prediction by the four-lncRNA signature within five years as the defining point in the training dataset. **C**. The four-lncRNA risk score distribution, overall survival of patients and heatmap of the four-lncRNA expression profiles in the training dataset.

To construct a prognostic signature, these four lncRNAs were analyzed using a multivariate Cox regression analysis in the training dataset with survival as the dependent variable and other clinical information as covariables. Then we constructed a prognostic signature by integrating the expression profiles of the four lncRNAs and corresponding estimated regression coefficient derived from above multivariate Cox regression analysis as follows: Risk score = (0.371 × expression value of *AC005682.5*) + (0.175 × expression value of *CTD-2231H16*.1) + (-0.251 × expression value of *CTB-92J24.2*) + (-0.232 × expression value of *RP11-727F15.13*). With the four-lncRNA signature, we calculated a risk score for each patient in the training dataset and ranked them according to increased risk score. Thus, 117 patients of the training dataset were classified into a high-risk group (*n* = 59) and a low-risk group (*n* = 58) using the median risk score (-1.12) as the cutoff point. The Kaplan-Meier analysis showed a significant difference in patients’ survival between the high-risk group and the low-risk group (log-rank test *p-value* = 8.94E-09; Figure 1A). Patients in the high-risk group had significantly shorter survival (median 5.60 months) than those in the low-risk group (median 7.52 months). To evaluate how well the four-lncRNA signature for predicting the 5-year survival, the time-dependent ROC curve analysis was carried out. The AUC for the four-lncRNA signature was 0.807 at the survival of five years (Figure 1B), demonstrating the competitive performance of the four-lncRNA signature for survival prediction in the training dataset. In the univariate Cox regression analysis of the training dataset, the four-lncRNA risk score were significantly associated with patients’ survival (*p-value* = 3.33E-05, HR = 23.141, 95% CI = 5.248-102.043; Table 2).

**Table 2:** Univariate and multivariate Cox regression analyses in each dataset

Variables	Univariate analysis			Multivariate analysis		
	HR	95% CI of HR	*P* value	HR	95% CI of HR	*P* value
Training dataset (n=117)						
Four-lncRNA risk score						
Low risk/High risk	23.141	5.248-102.043	3.33E-05	21.761	4.637-102.129	9.44E-05
Age						
≤65/>65	1.072	0.444-2.591	0.876	0.700	0.237-2.071	0.520
Gender						
Female/Male	0.786	0.304-2.031	0.620	0.909	0.331-2.498	0.853
Subtype						
Non-Papillary/Papillary	0.343	0.100-1.173	8.82E-02	0.290	0.060-1.401	0.123
Stage						
II	1 (reference)			1 (reference)		
III	2.408	0.598-9.701	0.217	0.246	0.031-1.946	0.184
IV	3.350	0.944-11.882	6.13E-02	1.369	0.335-5.591	0.662
**Testing dataset (n=117)**						
Four-lncRNA risk score						
Low risk/High risk	2.365	1.042-5.372	3.97E-02	2.459	1.067-5.666	3.46E-02
Age						
≤65/>65	1.148	0.479-2.754	0.756	1.333	0.549-3.240	0.525
Gender						
Female/Male	1.199	0.481-2.991	0.697	1.229	0.488-3.096	0.662
Subtype						
Non-Papillary/Papillary	0.507	0.175-1.473	0.212	0.633	0.213-1.885	0.411
Stage						
II	1 (reference)			1 (reference)		
III	1.680	0.515-5.484	0.390	1.479	0.422-5.180	0.541
IV	2.472	0.791-7.720	0.119	2.024	0.634-6.468	0.234
**Entire dataset (n=234)**						
Four-lncRNA risk score						
Low risk/High risk	5.581	2.839-10.972	6.18E-07	4.975	2.527-9.795	3.45E-06
Age						
≤65/>65	1.118	0.603-2.073	0.722	1.202	0.643-2.248	0.564
Gender						
Female/Male	0.994	0.516-1.915	0.985	1.145	0.587-2.233	0.691
Subtype						
Non-Papillary/Papillary	0.433	0.194-0.967	4.12E-02	0.553	0.242-1.268	0.162
Stage						
II	1 (reference)			1 (reference)		
III	2.070	0.840-5.099	0.114	1.070	0.383-2.992	0.898
IV	2.917	1.256-6.775	1.28E-02	1.866	0.774-4.497	0.165

The distribution of the risk score, overall survival and prognostic lncRNA expression profiles in 117 patients of the training dataset were showed in Figure 1C, ranked according to increased risk score. Patients with high-risk scores had higher mortality than patients with low-risk scores. For patients with high risk scores, the expression profiles of lncRNAs (*AC005682.5* and *CTD-2231H16.1*) are significantly up-regulated, while the remaining two lncRNAs (*CTB-92J24.2* and *RP11-727F15.13*) were down-regulated.

### Validation of the four-lncRNA signature for survival prediction in the testing dataset and entire dataset

**Figure 2:**
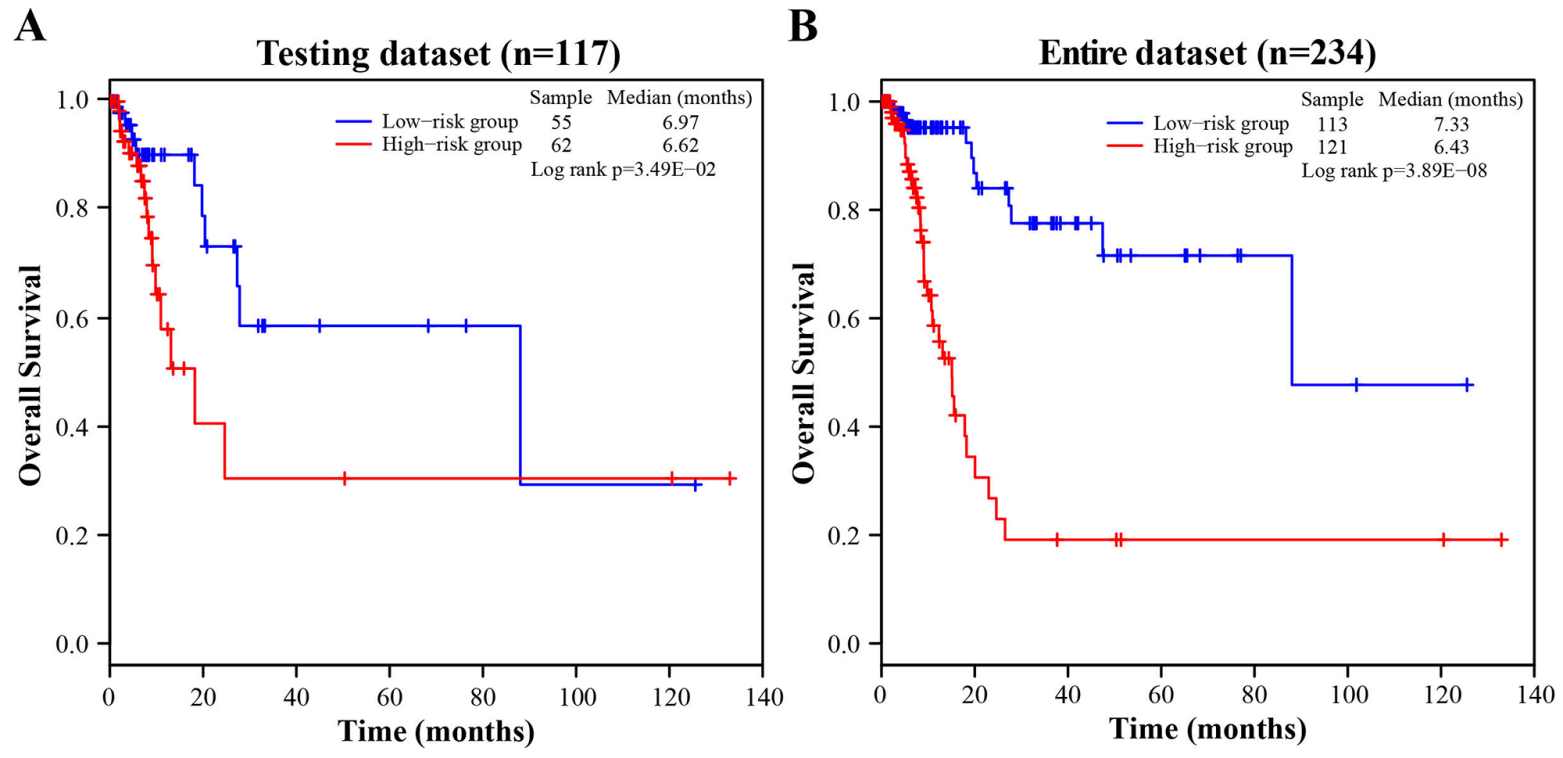
The Kaplan-Meier curves of overall survival between high-risk and low-risk patients in the testing and entire dataset **A**. The Kaplan-Meier curves for the testing dataset. **B**. The Kaplan-Meier curves for the entire dataset.

To validate the prognostic power of the four-lncRNA signature for survival prediction, 117 patients of the testing dataset were divided into a high-risk group (*n* = 62) and a low-risk group (*n* = 55) with the same lncRNA signature and cutoff point derived from the training dataset. In consistent with the findings in the training dataset, the result showed that a significantly different survival between the high-risk group and the low-risk group (log-rank test *p-value* = 3.49E-02, median 6.62 months *vs*. 6.97 months; Figure 2A). The AUC for the four-lncRNA signature was 0.656 at the survival of five years in the testing dataset. In the univariate Cox regression analysis of the testing dataset, the four-lncRNA risk score were significantly associated with patients’ survival (*p-value* = 3.97E-02, HR = 2.365, 95% CI = 1.042-5.372; Table 2).

When the four-lncRNA signature was further applied to the entire TCGA dataset, similar results were observed. As in the training and testing dataset, the four-lncRNA signature could also classify 234 patients of the entire dataset into a high-risk group (*n* = 121) and a low-risk group (*n =* 113) with significantly different survival (log-rank test *p-value* = 3.89E-08, median 6.43 months *vs*. 7.33 months; Figure 2B). In the entire dataset, the AUC for the four-lncRNA signature was 0.758 at the survival of five years. The univariate Cox regression analysis also demonstrated that the four-lncRNA risk score was significantly associated with patients’ survival in the entire dataset (*p-value* = 6.18E-07, HR = 5.58, 95% CI = 2.84-10.97; Table 2). Taken together, the above results demonstrated good reliability and reproducibility of the four-lncRNA signature for predicting BLCA patients’ survival.

### Independence of the four-lncRNA signature for survival prediction from other clinical variables

**Figure 3:**
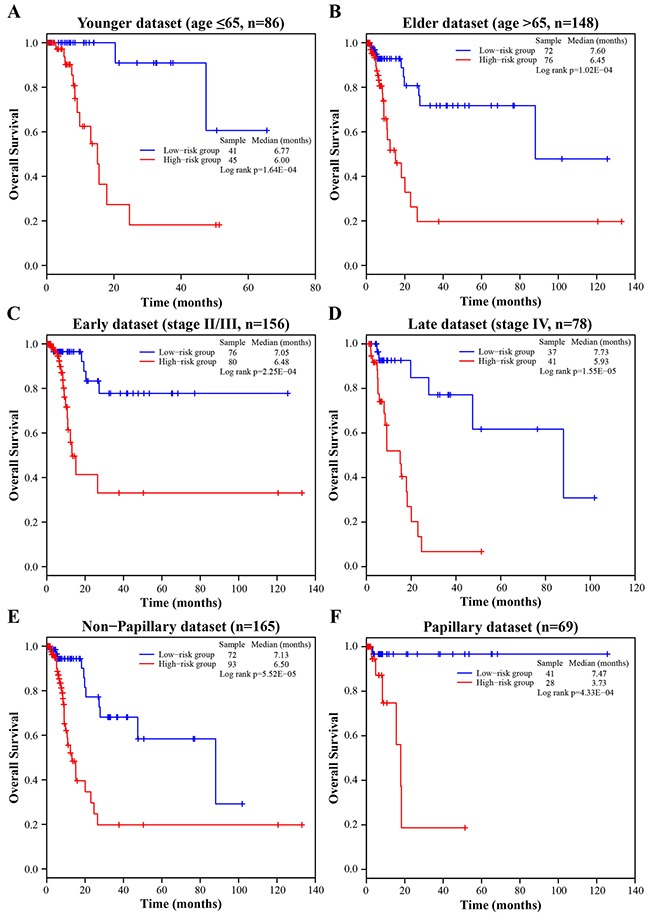
Survival analyses of all BLCA patients stratified by age, stage, tumor subtype with the four-lncRNA signature **A**. The Kaplan-Meier curves for the younger dataset (age ≤ 65, n = 86). **B**. The Kaplan-Meier curves for the elder dataset (age > 65, n = 148). **C**. The Kaplan-Meier curves for the early dataset (stage II/III, n = 156). **D**. The Kaplan-Meier curves for the late dataset (stage IV, n = 78). **E**. The Kaplan-Meier curves for the non-papillary dataset (subtype of non-papillary, n = 165). **F**. The Kaplan-Meier curves for papillary dataset (subtype of papillary, n = 69).

To evaluate whether the prognostic power of the four-lncRNA signature was independent of other clinical variables including age, gender, subtype and tumor stage, the multivariate Cox regression analyses were first carried out in each dataset. The results from the three datasets demonstrated that the four-lncRNA risk score was significantly associated with patients’ survival. Specifically, the four-lncRNA signature still maintained an independent association with survival after adjustment for other clinical variables in the training dataset (*p-value* = 9.44E-05, HR = 21.761, 95% CI = 4.637-102.129), testing dataset (*p-value* = 3.46E-02, HR = 2.459, 95% CI = 1.067-5.666) and entire dataset (*p-value* = 3.45E-06, HR = 4.975, 95% CI = 2.527-9.795; Table 2). Next, stratified analyses were then performed according to age, tumor stage and subtype, respectively. First, all 234 BLCA patients were stratified by the age (65 years old) into a younger dataset (*n* = 86) and an elder dataset (*n* = 148). The four-lncRNA signature could classify the younger dataset into a high-risk group (*n* = 45) and a low-risk group (*n* = 41) with significantly different survival (log-rank test *p-value* = 1.64E-04, median 6.00 months *vs*. 6.77 months; Figure 3A). Similarly, the four-lncRNA signature was also able to classify the elder dataset into a high-risk group (*n* = 76) and a low-risk group (*n* = 72) with significantly different survival (log-rank test *p-value* = 1.02E-04, median 6.45 months *vs*. 7.60 months; Figure 3B). Then all patients were further stratified by the tumor stage into an early dataset (stage II and stage III, *n* = 156) and a late dataset (stage IV, *n* = 78). Similar prognostic power of the four-lncRNA signature was significant in both the early dataset and late dataset. Patients in the early dataset were classified into a high-risk group (*n* = 80) with shorter survival and a low-risk group (*n* = 76) with longer survival (log-rank test *p-value* = 2.25E-04, median 6.48 months *vs*. 7.05 months; Figure 3C). Similar results were observed in the late dataset (log-rank test *p* = 1.55E-05, median 5.93 months *vs*. 7.73 months; Figure 3D). Finally, all patients were stratified by the subtype into a non-papillary dataset (*n* = 165) and a papillary dataset (*n* = 69). Significant differences in patients’ survival between the high-risk groups and the low-risk groups were also observed in the two datasets (log-rank test *p-value* = 5.52E-05, median 6.50 months *vs*. 7.13 months, Figure 3E; log-rank test *p-value* = 4.33E-04, median 3.73 months *vs*. 7.47 months, Figure 3F). The results of multivariate Cox regression analyses, together with the stratified analyses, demonstrated that the prognostic power of the four-lncRNA signature was independent of other clinical variables for survival prediction of patients with BLCA.

### Functional characteristics of the four prognostic lncRNAs

**Figure 4:**
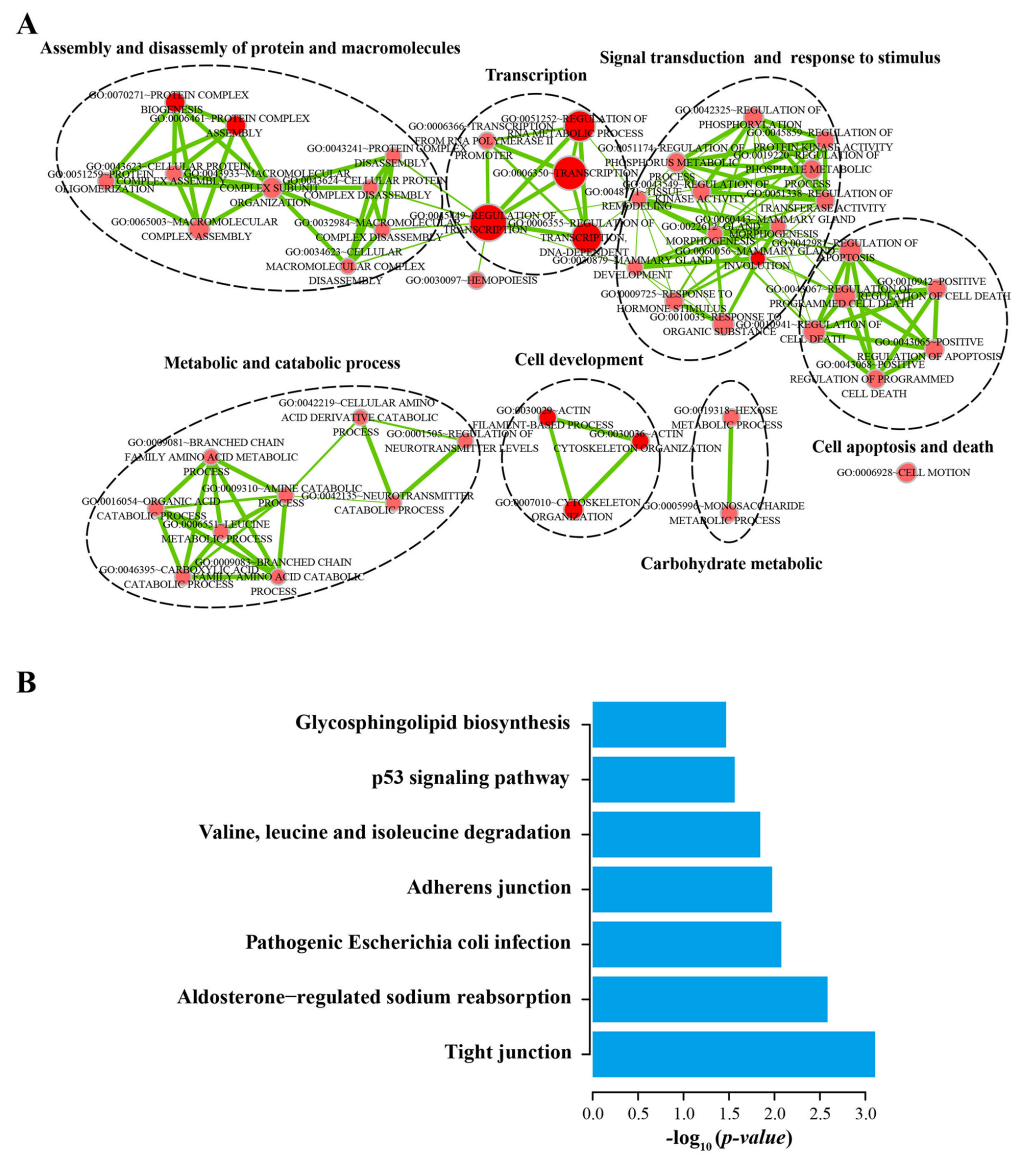
Functional enrichment analyses of the protein-coding genes co-expressed with the four prognostic lncRNAs **A**. The functional enrichment map of GO terms. *Each node* represents a GO term. An *edge* represents the overlap of the shared genes between connecting terms. *Node size* represents the number of gene in the GO terms. *Color intensity* is proportional to enrichment significance. **B.** Significantly enriched KEGG pathway.

To investigate potential functional roles of the four prognostic lncRNAs in BLCA tumorigenesis, we carried out functional enrichment analyses to predict their functions [44]. We first calculate Spearman correlation coefficients between lncRNAs and protein-coding genes by examining the paired lncRNA and the protein-coding gene expression profiles of 234 patients with BLCA. A total of 1405 protein-coding genes were significantly correlated with at least one of four prognostic lncRNAs (Spearman correlation coefficient > 0.40). Functional enrichment analyses of GO and KEGG pathways revealed that 1405 protein-coding genes were significantly enriched in 50 GO terms (a *p-value* of < 0.05 and an enrichment score of > 1.0) and 7 KEGG pathways (a *p-value* of < 0.05 and a fold enrichment of > 2.0). These functionally related GO terms were mainly organized into seven functional clusters including assembly and disassembly of protein and macromolecules, transcription, signal transduction and response to stimulus, cell apoptosis and death, metabolic and catabolic process, cell development, carbohydrate metabolic (Figure 4A). Seven significantly enriched KEGG pathways were observed including tight junction, aldosterone-regulated sodium reabsorption, pathogenic escherichia coli infection, adherens junction, valine, leucine and isoleucine degradation, p53 signaling pathway, glycosphingolipid biosynthesis (Figure 4B). These results of the functional enrichment analyses suggested that the four prognostic lncRNAs may participate in tumorigenesis through regulating or interacting protein-coding genes to affect known BLCA-related biological processes and pathways.

## DISCUSSION

Considerable efforts have been made during the past years to identify expression-based prognostic biomarkers for bladder cancer at protein-coding genes and miRNAs levels [45–47]. More recently, accumulated evidence indicates that dysregulated lncRNAs are implicated in various tumorigenesis processes including proliferation, invasion and apoptosis by acting as tumor oncogenes or suppressor, which has developed a new area for biomarkers. Additionally, plenty of aberrant lncRNA expression in multiple cancers was discovered by the transcriptional profiling analyses [48, 49], highlighting their potential roles as novel independent biomarkers for cancer prognosis. By now, several expression-based lncRNA signatures have been identified in glioblastoma, oesophageal squamous cell carcinoma, breast cancer, colorectal cancer, non-small cell lung cancer, multiple myeloma and ovarian cancer. Compared with protein-coding genes, lncRNAs exhibit greater tissue-, disease- and developmental stage-specific expression, and their expression is more closely related to the tumor status and biological functions [50–54]. Indeed, several lncRNAs, such as *HOTAIR*, *SPRY4-IT1*, *SUMO1P3* and *PANDAR* have been found to be associated with bladder prognosis [40, 43]. However, to date, the prognostic power of expression-based lncRNA signature for predicting survival in patients with BLCA has not yet been investigated.

Here, we performed a comprehensive analysis for lncRNA expression profiles and corresponding clinical information of BLCA patients in the training dataset, and identified four lncRNAs significantly associated with patients’ survival and constructed a four-lncRNA signature that can effectively predict survival of BLCA patients. Further ROC curve analysis demonstrated the competitive performance of the four-lncRNA signature for predicting 5-year survival in the training dataset. Then the prognostic power of the four-lncRNA signature was validated in an independent non-overlapping dataset and an entire dataset, demonstrated good reliability and reproducibility of the four-lncRNA signature for predicting BLCA patients’ survival. Next, we performed multivariate Cox regression analyses in each dataset to evaluate whether the prognostic power of the four-lncRNA signature was independently of other clinical variables, including age, gender, subtype and tumor stage. The four-lncRNA signature was demonstrated to still maintain an independent association with patients’ survival after adjustment for other clinical variables. In the stratified analyses, the four-lncRNA signature showed prognostic power for the younger dataset (age ≤ 65) and the elder dataset (age > 65), in which patients belonging to the two datasets could be classified into high-risk and low-risk groups with significant differences in patients’ survival. Similar prognostic power of the four-lncRNA signature was also significant in the early dataset (stage II and stage III) and the late dataset (stage IV), in which patients in the two datasets were able to classified into high-risk and low-risk groups with significantly different survival. Moreover, similar results were observed in the non-papillary dataset and the papillary dataset. Taken together, these results demonstrated that the prognostic power of the four-lncRNA signature was independent of other clinical variables for survival prediction of patients with BLCA.

Up to date, although more than tens of thousands of lncRNAs have been discovered in humans over the past few decades [55], only a handful of lncRNAs were functionally well-characterized and the functional study of lncRNAs remains in its infancy. Previous studies have suggested that lncRNAs participated in biological processes and pathways by regulating or interacting with protein-coding genes involved in the same processes, making it possible to infer lncRNA biological functions from their co-expressed protein-coding genes [44, 56, 57]. To detect the biological implication of the four prognostic lncRNAs in BLCA, we performed GO and KEGG functional enrichment analyses for co-expressed protein-coding genes. The results indicated the important functional roles of the four prognostic lncRNAs in tumorigenesis.

In summary, by performing a comprehensive analysis for lncRNA expression profiles and corresponding clinical information, our study identified four prognostic lncRNAs were significantly associated with BLCA patients’ survival and constructed a four-lncRNA signature that can effectively predict patients’ survival. The prognostic power of the four-lncRNA signature was independent of other clinical variables, and showed superior performance compared to known traditional clinical variables in a way. Our results demonstrated the four-lncRNA signature can function as novel independent biomarkers for BLCA prognosis and provided novel insights into understanding the underlying molecular mechanism of BLCA.

## MATERIALS AND METHODS

### BLCA datasets and patient information

The lncRNA expression profiles and corresponding clinical information of BLCA patients were obtained from The Cancer Genome Atlas (TCGA) data portal (up to May 27, 2016; https://gdc-portal.nci.nih.gov/). A total of 234 patients were enrolled in this study after removal of patients without available clinical information. Clinical information of BLCA patients used in this study, including age, gender, subtype and stage. More detailed clinical characteristics of all 234 BLCA patients in this study were listed in Table 3.

**Table 3:** Clinical characteristics of patients with BLCA in this study

Characteristics	Training dataset(n=117)	Testing dataset(n=117)	Entire dataset(n=234)
Vital status			
Alive	96 (82.1%)	91 (77.8%)	187 (79.9%)
Dead	21 (17.9%)	26 (22.2%)	47 (20.1%)
Age			
≤65	50 (42.7%)	36 (30.8%)	86 (36.8%)
>65	67 (57.3%)	81 (69.2%)	148 (63.2%)
Gender			
Female	31 (26.5%)	28 (23.9%)	59 (25.2%)
Male	86 (73.5%)	89 (76.1%)	175 (74.8%)
Subtype			
Non-papillary	82 (70.1%)	83 (70.9%)	165 (70.5%)
Papillary	35 (29.9%)	34 (29.1%)	69 (29.5%)
Stage			
II	41 (35.0%)	29 (24.8%)	70 (29.9%)
III	37 (31.6%)	49 (41.9%)	86 (36.8%)
IV	39 (33.3%)	39 (33.3%)	78 (33.3%)

### Acquisition of lncRNA expression profiles

The lncRNAs derived from TCGA and lncRNAs from GENCODE project [58] were cross-reference by Ensemble ID to reduce redundant. Then the lncRNA expression profiles were defined as those with an average RPKM ≥ 0.3 across all 234 BLCA patients. Finally, we obtained expression profiles of 12730 lncRNAs in 234 BLCA patients.

### Construction of a prognostic lncRNA signature

The lncRNAs expression profiles were normalized by log2 transformed. The association between the expression profiles of each lncRNA and patients’ survival was evaluated in the training dataset using a univariate Cox regression analysis with survival as the dependent variable. LncRNAs whose expression profiles were significantly associated with patients’ survival were identified (*p-value* < 0.001) as prognostic lncRNAs, and then subjected to a multivariate Cox regression analysis in the training dataset with survival as the dependent variable and other clinical information as covariables. Subsequently, a prognostic lncRNA signature was constructed based on a linear combination of the expression profiles of prognostic lncRNAs with weighted by the estimated regression coefficient as follows:

$$\text{Risk Score}(RS)=\sum_{i=1}^{N}\;({\text{Exp}}_{i}*{\text{Coe}}_{i})$$

where *N* is the number of prognostic lncRNAs, *Exp_i_* is the expression profiles of *lncRNA_i_*, and *Coe_i_* is the estimated regression coefficient of *lncRNA_i_* derived from the multivariate Cox regression analysis. The lncRNA signature could calculate a risk score for each patient. With the lncRNA signature, BLCA patients in each dataset were classified into high-risk and low-risk groups using the median risk score derived from the training dataset as a cutoff point.

### Statistical analysis

Differences in patients’ survival between the high-risk group and the low-risk group in each dataset were accessed by the Kaplan-Meier survival analyses, and compared by the two-sided log-rank test using the R package “survival” [59]. Furthermore, in order to evaluate whether the prognostic power of the four-lncRNA signature was independent of other clinical variables including age, gender, subtype and tumor stage, multivariate Cox regression and stratified analyses were carried out in each dataset with survival as the dependent variable, lncRNA risk score and other clinical variables as explanatory variables. Hazard ratios (HR) and 95% confidence intervals (CI) were calculated. The time-dependent receiver operating characteristic (ROC) curve analyses within five years as the defining points were performed using the R package “survivalROCR” [60], which has been widely used to evaluate the prognostic performance for survival prediction [61]. Area under the ROC curve (AUC) values were calculated from the ROC curves. All analyses were performed using R software and Bioconductor (version 3.3.0).

### Functional enrichment analyses

Spearman correlation coefficients were computed to evaluate co-expression relationships between prognostic lncRNAs and protein-coding genes. Functional enrichment analyses for the co-expressed protein-coding genes were carried out using the DAVID Bioinformatics Tool (version 6.7) [62, 63] limited to Gene ontology (GO) terms in the “Biological Process” (GOTERM-BP-FAT) and Kyoto encyclopedia of genes and genomes (KEGG) pathway categories with the human whole genome as the background. GO terms with a *p-value* of < 0.05 and an enrichment score of > 1.0, KEGG pathway with a *p-value* of < 0.05 and a fold enrichment of > 2.0 were considered as significantly enriched function annotations. Significant enrichment results were visualized and clustered based on the similar function using the Enrichment Map plugin [64] in Cytoscape (version 3.4.0) [65] and R package “goProfiles” [66].
